# In-Hospital Venous Thromboembolism and Pulmonary Embolism After Major Urologic Cancer Surgery

**DOI:** 10.1245/s10434-023-14246-0

**Published:** 2023-09-18

**Authors:** Cristina Cano Garcia, Stefano Tappero, Mattia Luca Piccinelli, Francesco Barletta, Reha-Baris Incesu, Simone Morra, Lukas Scheipner, Andrea Baudo, Zhe Tian, Benedikt Hoeh, Francesco Chierigo, Gabriele Sorce, Fred Saad, Shahrokh F. Shariat, Luca Carmignani, Sascha Ahyai, Nicola Longo, Derya Tilki, Alberto Briganti, Ottavio De Cobell, Paolo Dell’Oglio, Philipp Mandel, Carlo Terrone, Felix K. H. Chun, Pierre I. Karakiewicz

**Affiliations:** 1 Cancer Prognostics and Health Outcomes Unit, Division of Urology, University of Montréal Health Center, Montreal, QC Canada; 2 Department of Urology, University Hospital Frankfurt, Goethe University Frankfurt, Frankfurt am Main, Germany; 3 Department of Urology, Ospedale Policlinico San Martino, Genoa, Italy; 4 Department of Surgical and Diagnostic Integrated Sciences (DISC), University of Genova, Genoa, Italy; 5 Department of Urology, IEO European Institute of Oncology, IRCCS, Milan, Italy; 6 Unit of Urology/Division of Oncology, Gianfranco Soldera Prostate Cancer Lab, IRCCS San Raffaele Scientific Institute, Vita-Salute San Raffaele University, Milan, Italy; 7 Martini-Klinik Prostate Cancer Center, University Hospital Hamburg-Eppendorf, Hamburg, Germany; 8 Department of Neurosciences, Reproductive Sciences and Odontostomatology, Urology Unit, University of Naples Federico II, Naples, Italy; 9 Department of Urology, Medical University of Graz, Graz, Austria; 10 Department of Urology, IRCCS Ospedale Galeazzi-Sant’Ambrogio, Milan, Italy; 11 Department of Urology, IRCCS Policlinico San Donato, Milan, Italy; 12 Department of Urology, ASST Grande Ospedale Metropolitano Niguarda, Milan, Italy; 13 Department of Urology, Comprehensive Cancer Center, Medical University of Vienna, Vienna, Austria; 14 Department of Urology, Weill Cornell Medical College, New York, NY USA; 15 Department of Urology, University of Texas Southwestern, Dallas, TX USA; 16 Hourani Center of Applied Scientific Research, Al-Ahliyya Amman University, Amman, Jordan; 17 Department of Urology, University Hospital Hamburg-Eppendorf, Hamburg, Germany; 18 Department of Urology, Koc University Hospital, Istanbul, Turkey; 19 Department of Urology, Netherlands Cancer Institute-Antoni van Leeuwenhoek Hospital, Amsterdam, The Netherlands; 20 Interventional Molecular Imaging Laboratory, Department of Radiology, Leiden University Medical Center, Leiden, The Netherlands

## Abstract

**Background:**

This study aimed to test for temporal trends of in-hospital venous thromboembolism (VTE) and pulmonary embolism (PE) after major urologic cancer surgery (MUCS).

**Methods:**

In the Nationwide Inpatient Sample (NIS) database (2010–2019), this study identified non-metastatic radical cystectomy (RC), radical prostatectomy (RP), radical nephrectomy (RN), and partial nephrectomy (PN) patients. Temporal trends of VTE and PE and multivariable logistic regression analyses (MLR) addressing VTE or PE, and mortality with VTE or PE were performed.

**Results:**

Of 196,915 patients, 1180 (1.0%) exhibited VTE and 583 (0.3%) exhibited PE. The VTE rates increased from 0.6 to 0.7% (estimated annual percentage change [EAPC] + 4.0%; *p* = 0.01). Conversely, the PE rates decreased from 0.4 to 0.2% (EAPC − 4.5%; *p* = 0.01). No difference was observed in mortality with VTE (EAPC − 2.1%; *p* = 0.7) or with PE (EAPC − 1.2%; *p* = 0.8). In MLR relative to RP, RC (odds ratio [OR] 5.1), RN (OR 4.5), and PN (OR 3.6) were associated with higher VTE risk (all *p* < 0.001). Similarly in MLR relative to RP, RC (OR 4.6), RN (OR 3.3), and PN (OR 3.9) were associated with higher PE risk (all *p* < 0.001). In MLR, the risk of mortality was higher when VTE or PE was present in RC (VTE: OR 3.7, PE: OR  4.8; both *p* < 0.001) and RN (VTE: OR 5.2, PE: OR  8.3; both *p* < 0.001).

**Conclusions:**

RC, RN, and PN predisposes to a higher VTE and PE rates than RP. Moreover, among RC and RN patients with either VTE or PE, mortality is substantially higher than among their VTE or PE-free counterparts.

Advanced age, surgery, and presence of malignant disease represent major risk factors for venous thromboembolism (VTE).^1^ Within VTE, pulmonary embolism (PE) represents a rare but dreaded major complication. Patients undergoing major urologic cancer surgery (MUCS) are at a high risk for VTE.^2^

Specific guidelines for thromboprophylaxis after urologic surgery according to the risk of VTE and major bleeding were developed.^3–5^ However, the contemporary rates of in-hospital VTE and PE in the four most frequent MUCSs, namely, radical cystectomy (RC), radical prostatectomy (RP), radical nephrectomy (RN), and partial nephrectomy (PN) are unknown, especially when population-based cohorts are used for data extraction. Moreover, it also is unknown to what extent VTE or PE is associated with contemporary in-hospital mortality and if differences in the magnitude of this association are observed when the four MUCSs of interest are analyzed.

We addressed these knowledge gaps and hypothesized that comparable rates of VTE and PE for patients undergoing MUCS may be expected over time. Moreover, we hypothesized that moderate differences in association of VTE and mortality, as well as in association of PE and mortality, exist between the four MUCSs. To test these hypotheses, we relied on the Nationwide Inpatient Sample (NIS) database 2010–2019.

## Patients and Methods

### Patients

From the NIS 2010–2019, we selected patients 18 years old or older who underwent MUCS according to specific International Classification of Diseases (ICD)-9 and ICD-10 coding system (Table 1). Patients with metastatic cancer and those who underwent inferior vena cava thrombectomy (ICD-Procedure Coding System [PCS]-9: 380.7; ICD- PCS-1006C.00ZZ, 06C.03ZZ, 06C.04ZZ) were excluded. Further exclusion criteria ruled out unknown complications and survival data.^6^

**Table 1:** Baseline characteristics of 196,915 patients undergoing major urological cancer surgery in NIS 2010–2019 with versus without in-hospital venous thromboembolism (VTE)

Characteristic	With VTE	Without VTE	*p* Value^b^
	(*n* = 1,180, 1%)^a^	(*n* = 195,735, 99%)^a^	
	*n* (%)	*n* (%)	
Median age: years (IQR)	67 (60–74)	63 (57–69)	< 0.001
Female sex	271 (23)	27,543 (14)	< 0.001
Median hospital stay: days (IQR)	10 (6–17)	2 (1–4)	< 0.001
Charlson Comorbidity Index			< 0.001
0	584 (49)	149,923 (70)	
1	274 (22)	29,891 (15)	
2	88 (8)	5,489 (3)	
≥3	234 (20)	10,432 (5)	
Neoadjuvant chemotherapy	49 (4.2)	2,284 (1,2)	< 0.001
Smoking habit	299 (25)	38,817 (20)	< 0.001
Obesity	215 (18)	24,286 (12)	< 0.001
Race/ethnicity			0.09
Caucasian	846 (72)	136,407 (70)	
African–American	144 (12)	22,204 (11)	
Hispanic	72 (6)	13,924 (7)	
Others	118 (10)	23,200 (12)	
Insurance status			<0.001
Private	389 (33)	95,526 (50)	
Medicaid	74 (6)	9,229 (5)	
Medicare	665 (56)	78,451 (40)	
Others	52 (4)	9,529 (5)	
Income			0.05
First quartile	315 (27)	46,698 (24)	
Second quartile	293 (25)	46,693 (24)	
Third quartile	284 (24)	50,359 (26)	
Fourth quartile	288 (24)	51,985 (27)	
High annual hospital volume	227 (19)	39,310 (20)	0.5
Region			0.03
Northeast	258 (22)	38,070 (19)	
Midwest	302 (26)	46,558 (24)	
South	406 (34)	73,005 (37)	
West	214 (18)	38,102 (19)	
Teaching hospital	936 (79)	146,773 (75)	< 0.001

Abbreviations: NIS = Nationwide Inpatient Sample; IQR = interquartile range

^a^VTE disease codes: ICD-9-CM 453.4, 453.40, 453.41, 453.42, 453.8, 453.9, 415.x, 41511, 41512, 41519 and ICM-10-CM I82.210, I82.220, I82.23, I82.290, I82.4.xx, I82.6.xx, I82.A1.xx, I82.B1.xx, I82.C1.xx, I82.8.xx, I82.890, I82.90, I26.xx, I28.x.

^b^Wilcoxon rank sum test; Pearson’s chi-square test

Due to the anonymously coded design of the NIS, study-specific ethics approval was waived by the institutional review board.

### Statistical Analysis

First, we evaluated temporal trends of in-hospital VTE as well as in-hospital mortality with VTE in the NIS. Moreover, patient characteristics, namely, age of 69 years or older (75th percentile), distribution of Charlson Comorbidity Index (CCI), and a body mass index (BMI) rate of 30 kg/m^2^ or higher (obesity), for patients undergoing MUCS during the study span were analyzed. Estimated annual percentage changes (EAPCs) were tested with least squares linear regression. Second, multivariable logistic regression analyses tested for differences in VTE rates between the four MUCSs of interest. Third, uni- and multivariable logistic regression models tested for differences in prediction of mortality with VTE in the four MUCSs. Finally, the aforementioned method was reapplied in analyses addressing PE rates and mortality with PE. All uni- and multivariable analyses were fitted after adjustment for clustering at the hospital level using generalized estimation equation methodology.

In all statistical analyses R software environment for statistical computing and graphics (R version 4.1.3, R Foundation for Statical Computing, Vienna, Austria) was used.^7^ All tests were two sided, with a significance level set at a *p* value lower than 0.05.

## Results

### Descriptive Characteristics

Of the 196,915 study patients, 1180 (1.0%) exhibited VTE after MUCS. The VTE patients were older at diagnosis (67 vs 63 years; *p* < 0.001), were more frequently female (23% vs 14%; *p* < 0.001), exhibited a higher CCI (CCI 1 [22% vs 15%], CCI 2 [8% vs 3%], CCI ≥3 [20% vs 5%]; *p* < 0.001), were more often treated with neoadjuvant chemotherapy (4.2% vs 1.2%; *p* < 0.001), were more frequently smokers (25% vs 20%; *p* < 0.001), exhibited a higher rate of obesity (18% vs 12%; *p* < 0.001) and were more frequently treated in teaching hospitals (79% vs 75%; *p* < 0.001) than their no-VTE counterparts. Conversely, the VTE patients had private insurance status less frequently (33% vs 50%; *p* < 0.001; Table 1).

In analyses focusing on PE, 583 (0.3%) of 196,915 study patients had PE. The PE patients differed from the no-PE patients with regard to age, female sex, hospital length of stay, CCI, neoadjuvant chemotherapy, smoking habit, obesity, and insurance in a fashion similar to that recorded in VTE and no-VTE patient comparisons (Table 2).

**Table 2:** Baseline characteristics of 196,915 patients undergoing major urologic cancer surgery in NIS 2010–2019 with versus without in-hospital pulmonary embolism (PE)

Characteristic	With PE	Without PE	*p* Value^b^
	(*n* = 583, 0.3%)^a^	(*n* = 196,332, 99.7%)^a^	
	*n* (%)	*n* (%)	
Median age: years (IQR)	66 (58–73)	63 (57, 69)	< 0.001
Female sex	120 (21)	27,694 (14)	< 0.001
Median hospital stay: days (IQR)	10 (7–16)	2 (1–4)	< 0.001
Charlson Comorbidity Index			< 0.001
0	299 (51)	150,208 (77)	
1	131 (22)	30,034 (15)	
2	44 (8)	5,533 (3)	
≥3	109 (19)	10,557 (6)	
Neoaduvant chemotherapy	23 (4)	2,310 (1.2)	< 0.001
Smoking habit	140 (24)	38,976 (20)	0.012
Obesity	106 (18)	24,395 (12)	< 0.001
Race/ethnicity			0.003
Caucasian	417 (72)	136,836 (70)	
African–American	80 (14)	22,268 (11)	
Hispanic	29 (5)	13,967 (7)	
Others	57 (10)	23,261 (12)	
Insurance status			< 0.001
Private	192 (33)	98,723 (50)	
Medicaid	38 (7)	9,265 (5)	
Medicare	327 (56)	78,789 (40)	
Others	26 (4)	9,555 (5)	
Income			0.1
First quartile	159 (27)	46,854 (24)	
Second quartile	143 (25)	46,843 (24)	
Third quartile	138 (24)	50,505 (26)	
Fourth quartile	143 (25)	52,130 (27)	
High annual hospital volume	100 (17)	39,437 (20)	0.3
Region			0.2
Northeast	133 (23)	38,195 (19)	
Midwest	133 (23)	46,727 (24)	
South	199 (34)	73,212 (37)	
West	118 (20)	38,198 (19)	
Teaching hospital	447 (77)	147,262 (75)	0.4

Abbreviations: NIS = Nationwide Inpatient Sample; IQR = interquartile range

^a^PE disease codes: ICD-9-CM 415.x, 41511, 41512, 41519 and ICD-10-CM I26.xx, I28.xx

^b^Wilcoxon rank sum test; Pearson’s chi-square test

### Annual Trends in Venous Thromboembolism and Mortality with Venous Thromboembolism Among Patients Undergoing Major Urologic Cancer Surgery

**Fig. 1:**
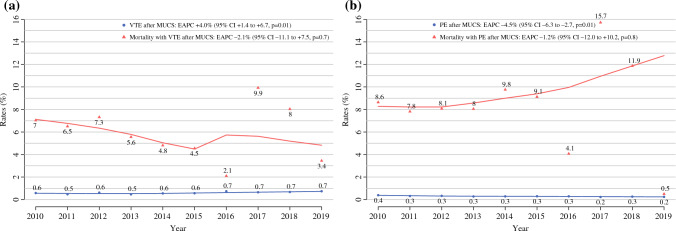
**a** Rates of in-hospital venous thromboembolism (VTE) and in-hospital mortality with VTE and **b** rates of in-hospital pulmonary embolism (PE) and in-hospital mortality with PE for patients undergoing major urologic cancer surgery (MUCS) in the Nationwide Inpatient Sample (NIS) database from 2010 to 2019

The rates of VTE for MUCS patients increased from 0.6 to 0.7%, between 2010 and 2019 (EAPC + 4.0%; 95% confidence interval [CI] 1.4% to 6.7%; *p* = 0.01). However, no difference in mortality with VTE was observed (EAPC − 2.1%; 95% CI − 11.1% to 7.5%; *p* = 0.7) (Fig. 1a).

### Annual Trends in Pulmonary Embolism and Mortality with Pulmonary Embolism Among Patients Undergoing Major Urologic Cancer Surgery

The rates of PE after MUCS decreased from 0.4 to 0.2% between 2010 and 2019 (EAPC − 4.5%; 95% CI − 6.3% to 2.7%; *p* = 0.01). Conversely, no difference in mortality with PE was observed (EAPC − 1.2%; 95% CI − 12.0% to 10.2%; *p* = 0.8; Fig. 1b).

### Annual Trends in the Charlson Comorbidity Index, Obesity, and Age 69 Years or Older Among Patients Undergoing Major Urologic Cancer Surgery

**Fig. 2:**
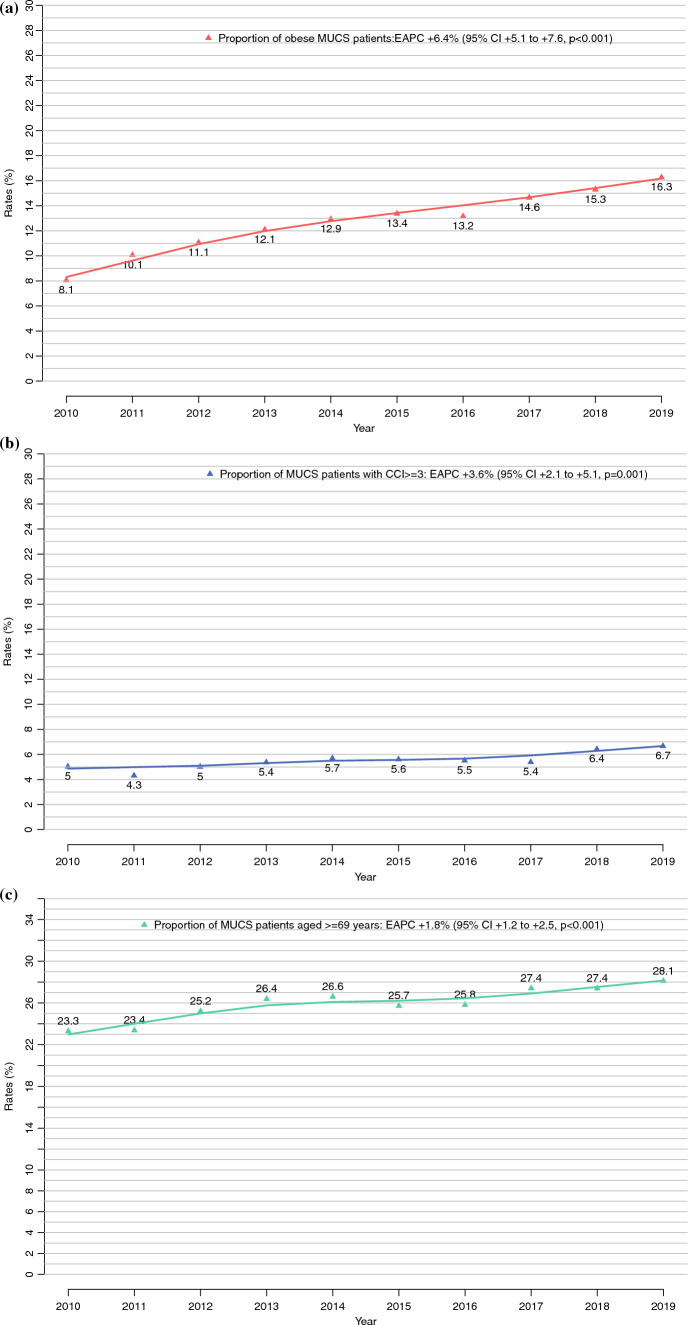
**a** Proportion of obese patients (body mass index ≥30 kg/m^2^) undergoing major urologic cancer surgery (MUCS) in the Nationwide Inpatient Sample (NIS) database from 2010 to 2019. **b** Proportion of patients with a Charlson Comorbidity Index (CCI) ≥3 undergoing MUCS in the Nationwide Inpatient Sample (NIS) database from 2010 to 2019. **c** Proportions of patients age ≥69 years undergoing MUCS in the Nationwide Inpatient Sample (NIS) database from 2010 to 2019

The rate of obesited patients increased from 8.1 to 16.3% (EAPC + 6.4%; 95% CI 5.1–7.6%; *p* < 0.0001) between 2010 and 2019. Similarly, the rate of CCI ≥3 increased from 5.0 to 6.7% (EAPC + 3.6; 95% CI 2.1–5.1; *p* = 0.001) during the same time period. Additionally, the rates for the patients 69 years or older increased from 23.3 to 28.1% (EAPC + 1.8%; 95% CI 1.2–2.5%; *p* < 0.001; Fig. 2a–c).

### Distribution of Venous Thromboembolism and Pulmonary Embolism Rates After Major Urologic Cancer Surgery

Of 196,915 study patients, 14,362 (7.3%) underwent RC versus 43,885 (22.2%) RN versus 25,211 (12.8%) PN versus 113,457 (57.6%) RP. Specifically, the rates for the minimally invasive surgical approach were highest in RP (75%), followed by PN (54.6%), RC (28.2%), and RN (20.5%).

The VTE rates after MUCS were highest for RC (377/14,362, 2.6%), followed by RN (472/43,885, 1.1%), PN (160/25,211, 0.6%), and RP (171/113,457, 0.2%). The differences in VTE rates between open and minimally invasive MUCS also were recorded. Specifically, the VTE rates were 2.7% versus 2.3% after open versus minimally invasive RC, 1.1% versus 0.9% after open versus minimally invasive RN, 0.8% versus 0.5% after open versus minimally invasive PN, and 0.2% versus 0.1% after open versus minimally invasive RP.

The PE rates after MUCS were highest for RC (170/14,362, 1.2%), followed by RN (203/43,885, 0.5%), PN (107/25,211, 0.4%) and RP (103/113,457, 0.1%). The differences in PE rates between open and minimally invasive MUCS also were recorded. Specifically, the PE rates were 1.2% versus 1.1% after open vs. minimally invasive RC, 0.5% versus 0.4% after open versus minimally invasive RN, 0.5% versus 0.3% after open versus minimally invasive PN, and 0.2% versus 0.007% after open versus minimally invasive RP (Table 3).

**Table 3:** In-hospital venous thromboembolism (VTE) and pulmonary embolism (PE) of 196,915 patients undergoing major urologic cancer surgery (radical cystectomy, radical nephrectomy, partial nephrectomy, radical prostatectomy) in NIS 2010–2019

Surgery	Overall (*n* = 196,915, 100%)	With VTE	With PE
	*n* (%)	*n* (%)	*n* (%)
Radical cystectomy (RC)^a^	14,362 (7.3)	377/14,362 (2.6)	170/14,362 (1.2)
Open RC	10,319 (71.8)	283/10,319 (2.7)	124/10,319 (1.2)
Minimally invasive RC	4,043 (28.2)	94/4,043 (2.3)	46/4,043 (1.1)
Radical nephrectomy (RN)^b^	885 (22.2)	472/43,885 (1.1)	203/43,885 (0.5)
Open RN	34,877 (79.5)	393/34,877 (1.1)	169/34,877 (0.5)
Minimally invasive RN	9,008 (20.5)	79/9,008 (0.9)	34/9,008 (0.4)
Partial nephrectomy (PN)^c^	25,211 (12.8)	160/25,211 (0.6)	107/25,211 (0.4)
Open PN	11,425 (45.3)	92/11,425 (0.8)	61/11,425 (0.5)
Minimally invasive PN	13,786 (54.6)	68/13,786 (0.5)	46/13,786 (0.3)
Radical prostatectomy (RP)^d^	113,457 (57.6)	171/113,457 (0.2)	103/113,457 (0.09)
Open RP	24,938 (22.0)	60/24,938 (0.2)	43/24,938 (0.2)
Minimally invasive RP	88,519 (78.0)	111/88,519 (0.1)	60/88,519 (0.07)

Abbreviations: NIS = Nationwide Inpatient Sample

^a^RC surgical-site codes: ICD-9-PCS 577, 577.1, 577.9 and ICD-10-PCS 0TT.B0ZZ, 0TR.B07Z, 0TT.B3ZZ, 0TT.B4ZZ, 0TT.B8ZZ, 0TR.B47Z, 0TT.B7ZZ, 0TR.B07Z

^b^RN surgical-site codes: ICD-9-PCS 555, 555.1, 555.2, 555.4 and ICD-10-PCS 0TT.00ZZ, 0TT.04ZZ, 0TT.10ZZ, 0TT.14ZZ, 00TT.20ZZ, 00TT.24ZZ

^c^PN surgical-site codes: ICD-9-PCS 554 and ICD-10-PCS 0TB.00ZZ, 0TB.03ZZ, 0TB.04ZZ, 0TB.07ZZ, 0TB.08ZZ, 0TB.10ZZ, 0TB.13ZZ, 0TB.14ZZ, 0TB.17ZZ, 0TB.18ZZ

^d^RP surgical-site codes: ICD-9-PCS 604, 605, 606.2 and ICD-10-PCS 0VT.00ZZ, 0VT.04ZZ, 0VT.07ZZ, 0VT.08ZZ, 0V5.00ZZ, 0V5.03ZZ, 0V5.04ZZ

### Multivariable Logistic Regression Models Predicting Venous Thromboembolism After Major Urologic Cancer Surgery Relative to Radical Prostatectomy

In multivariable logistic regression models addressing VTE, the RC (odds ratio [OR], 4.9; 95% CI 3.8–6.4; *p* < 0.001), RN (OR, 4.5; 95% CI 3.5–5.7; *p* < 0.001), and PN (OR, 3.6; 95% CI 2.7–4.7; *p* < 0.001) patients exhibited a higher risk of VTE than their RP counterparts (Table 4a).

**Table 4:** (a) Multivariable logistic regression models predicting in-hospital venous thromboembolism (VTE) after major urologic cancer surgery (MUCS). (b) Multivariable logistic regression models predicting in-hospital pulmonary embolism (PE) after major urologic cancer surgery (MUCS)

Characteristic	OR	95% CI	*p* Value
(a)			
Major urologic cancer surgery			
Radical prostatectomy	Reference	–	–
Radical cystectomy	4.9	3.8–6.4	< 0.001
Radical nephrectomy	4.5	3.5–5.7	< 0.001
Partial nephrectomy	3.6	2.7–4.7	< 0.001
(b)			
Major urologic cancer surgery			
Radical prostatectomy	Reference	–	–
Radical cystectomy	4.4	3.1–6.2	< 0.001
Radical nephrectomy	3.2	2.3–4.5	< 0.001
Partial nephrectomy	3.9	2.8–5.4	< 0.001

Abbreviations: OR = odds ratio; CI = confidence interval

Moreover, in multivariable logistic regression models addressing VTE, obesity was associated with a higher risk of VTE (OR, 1.3; 95% CI 1.1–1.6; *p* < 0.001; not shown in Table 4a).

### Multivariable Logistic Regression Models Predicting Pulmonary Embolism After Major Urologic Cancer Surgery Relative to Radical Prostatectomy

In multivariable logistic regression models addressing PE, the RC (hazard ratio [HR], 4.4; 95% CI 3.1–6.2; *p* < 0.001), RN (HR, 3.2; 95% CI 2.3–4.5; *p* < 0.001), and PN (HR, 3.9; 95% CI 2.8–5.4; *p* < 0.001) patients exhibited a higher risk of PE than their RP counterparts (Table 4b).

Moreover, in multivariable logistic regression models addressing PE, obesity was associated with a higher risk of VTE (OR, 1.4; 95% CI 1.1–1.8; *p* < 0.001, not shown in Table 4b).

### Multivariable Logistic Regression Models Predicting Mortality with Venous Thromboembolism After Major Urologic Cancer Surgery

In multivariable logistics regression models, the presence of VTE was associated with a 3.7-fold higher mortality rate in RC (95% CI 2.3–5.8; *p* < 0.001) and a 5.2-fold higher mortality rate in RN (95% CI 3.1–8.8; *p* < 0.001). Due to the limited event rate for PN and RP, multivariable analyses were not performed (Table 5a).

**Table 5:** Logistic regression analyses predicting (a) in-hospital mortality with venous thromboembolism (VTE) relative to in-hospital mortality without VTE (reference) and (b) in-hospital mortality with pulmonary embolism (PE) relative to in-hospital mortality without PE (reference) after major urologic cancer surgery (MUCS)

	(a) Venous thromboembolism (VTE)					(b) Pulmonary embolism (PE)				
	Mortality with VTE (%)	Univariable		Multivariable^a^		Mortality with PE (%)	Univariable		Multivariable^a^	
		OR (95% CI)	*p* Value	OR (95% CI)	*p* Value		OR (95% CI)	*p* Value	OR (95% CI)	*p* Value
Radical cystectomy	13.1	6.0 (4.0–8.9)	< 0.001	3.7 (2.3–5.8)	< 0.001	8.0	7.9 (4.8–13.0)	< 0.001	4.9 (2.7–8.6)	< 0.001
Radical nephrectomy	11.2	12.4 (8.4–18.2)	< 0.001	5.2 (3.1–8.8)	< 0.001	7.1	18.1 (11.1–29.4)	< 0.001	8.3 (4.2–16.5)	< 0.001
Partial nephrectomy	13.4	24.5 (10.9–54.5)	< 0.001	–	11.5	32.1 (13.7–76.5)		–		
Radical prostatectomy	9.6	72.6 (28.7–183.0)	< 0.001	–	7.7	95.4 (33.7–269.9)		–		

^a^Covariables: age, year of diagnosis, length of stay, sex, race/ethnicity, smoking habit, obesity, annual hospital volume, institutional teaching status, CCI, region, surgical approach (minimally invasive vs open), lymph node dissection, neoadjuvant chemotherapy

### Multivariable Logistic Regression Models Predicting Mortality with Pulmonary Embolism After Major Urologic Cancer Surgery

In the multivariable logistics regression models, the presence of PE was associated with a 4.9-fold higher mortality rate in RC (95% CI 2.7–8.6; *p* < 0.001) and an 8.3-fold higher mortality rate in RN (95% CI 4.2–16.5; *p* < 0.001). Due to the limited event rate in PN and RP, multivariable analyses were not performed (Table 5b).

## Discussion

We examined contemporary trends in rates of in-hospital VTE and PE in the four most frequent MUCSs (RC, RP, RN, and PN) in a population-based cohort in NIS 2010–2019. We made several important observations.

First, we identified 1180 contemporary NIS 2010–2019 patients with in-hospital VTE when all four MUCSs were examined (RC, RN, PN, RP). The risk of VTE is non-negligible after MUCS.^3^ It represents a life-threating complication after most cancer surgeries, including the four examined MUCSs.^8–10^ However in the current study, the presence of VTE was identified in only 1% (*n* = 1180) of all the patients. Moreover, we identified 583 contemporary MUCS patients (0.3%) with PE. Data addressing rates of PE without combining it with deep vein thrombosis (DVT) are limited due to the rarity of PE after MUCS.^11^ Consequently, the current rate of PE cannot be directly compared with previous reports originating from population-based data repositories because such contemporary reports focusing on in-hospital PE rates do not exist. Therefore, population-based data repositories such as the NIS or the National Surgical Quality Improvement Program (NSQIP) are essential to study rare events associated with elevated fatality rates, especially when multivariable analyses are required.

Second, we observed a statistically significant increase in in-hospital VTE rates after MUCS (EAPC + 4.0%; *p* = 0.01). However, this increase was only marginal at best (from 0.6 to 0.7%) and may have no bearing in individual patient considerations. The recorded increase in in-hospital VTE rates may also suggest that more high-risk patients underwent MUCS. Indeed, the proportion of obese patients (BMI ≥30 kg/m^2^) increased from 8.1 to 16.3% (EAPC + 6.4%; *p* < 0.001). Moreover, the proportion of patients with a CCI of 3 or higher increased from 5.0 to 6.7% (EAPC + 3.6; *p* = 0.001). Finally, the proportion of MUCS patients age 69 years or older also increased from 23.3 to 28.1% (EAPC + 1.8%; *p* < 0.001). All three variables, namely, obesity (BMI ≥30 kg/m^2^), CCI (≥ 3), and advanced age (≥ 69 years) represent VTE risk factors.^1,12–15^ Specifically, obesity, which represents a potentially modifiable risk factor, was associated with higher risk of VTE (OR 1.3; *p* < 0.001) and PE (OR 1,4; *p* = 0.003) in the current study. Despite the consistent increase in VTE risk after MUCS, the VTE rate did not increase in a clinically meaningful fashion (from 0.6 to 0.7%). Similarly, mortality with VTE after MUCS did not increase (*p* = 0.8). These observations may be indicative of an improvement in quality of care over time.^16–19^ Therefore, it may be hypothesized that despite a higher patient risk profile and higher VTE rates, current VTE prevention and management strategies did not result in a mortality increase.

Third, we observed a statistically significant decrease in in-hospital PE rates after MUCS (EAPC − 4.5%; 95% CI − 6.3 to − 2.7%; *p* = 0.01). Although the absolute PE rate decrease was marginal at best (from 0.4 to 0.2%) and may have no bearing in individual patient considerations, a statistically significant decrease in such life-threatening complication is clearly encouraging. Consequently, as for VTE, such a decrease may be indicative of an improvement in quality of care over time, especially in light of the aforementioned increases in risk factors for VTE and vascular events in general.^16–19^

Fourth, we observed important differences in in-hospital VTE rates between the four MUCSs (RC versus RN versus PN versus RP). The rate of VTE was highest after RC (2.6%), followed by RN (1.1%), then PN (0.6%), and finally was lowest for RP (0.2%). In multivariable analyses addressing VTE rate and quantifying the magnitude of the differences between the four MUCSs, remarkably similar ORs were recorded for each of the three MUCSs that were compared with RP (OR of 4.9 for RC versus 4.5 for RN versus 3.6 for PN). This observation suggests that each of these three MUCSs (RC, RN, PN) should be considered as an equally important VTE risk factor. Consequently, RC, RN, and PN may be given virtually the same consideration in VTE strategies. The current results cannot be directly compared with previous reports because they did not focus on in-hospital VTE rates. Moreover, previous reports did not compare VTE risk after RC, RN, or PN relative to RP, which is known for its lowest VTE rate. Similarly, when PE rates were considered, remarkably similar ORs were recorded for the three MUCSs compared with RP (OR of 4.4 for RC versus 3.3 for RN versus 3.9 for PN).

Fifth, we have provided the most contemporary relative rates of mortality with VTE and PE. The specific multivariable increase in mortality with VTE was 3.7-fold for RC and 5.2-fold for RN. Similarly, the specific increase in mortality with PE was 4.9-fold for RC and 8.3-fold for RN. Unfortunately, multivariable rates could not be computed for PN and RP due to insufficient numbers of observations. The current findings cannot be directly compared with previous reports. To the best of our knowledge, we are the first to report the association between mortality and VTE in multivariable analyses for RC and RN patients.

Taken together, we observed increased rates of known VTE risk factors such as increased rates of obesity, CCI of 3 or higher, and age of 69 years or older. However, no substantial increase in VTE and PE rates and no increase in mortality with VTE or with PE were observed. Consequently, it may be postulated that VTE prevention and management strategies have improved over time. The same hypothesis may be proposed for PE. Moreover, it is also of interest to know that VTE risk was remarkably similar after RC (OR 5.1), RN (OR 4.5), and PN (OR 3.6) relative to RP, even after the most detailed multivariable adjustments. Based on these findings, clinicians should not underestimate the risk of VTE, even after RN and PN, which are considered as lower VTE risk interventions.^3,4^ Finally even after most detailed multivariable adjustments, the presence of VTE increased the mortality risk after RC in fourfold fashion, and after RN in fivefold fashion. Similarly, even after the most detailed multivariable adjustments, the presence of PE increased mortality risk after RC in fivefold fashion and after RN in eightfold fashion. These observations illustrate that even in contemporary MUCS patients, whose prophylactic and therapeutic interventions are standard of care, the increase in mortality is very substantial, and assessment of VTE status and of PE status even more so is crucial. This consideration applies not only to open RC and open RN, but also to minimally invasive RC and minimally invasive RN, which may be considered a lower-risk surgical approach by some.^12,13,20,21^

Despite its novelty, the current study had several limitations. First, we relied on the large-scale retrospective NIS database. All previous large-scale reports that addressed this topic also were retrospective.^12,22–24^ However, the current study relied on the most contemporary cohort (2010–2019), for which higher rates of minimally invasive MUCS, especially for RC, were observed. Second, our analyses relied on a limited number of events. Specifically, for PN and RP, multivariable analyses were not possible due to insufficient numbers of observations. Consequently, ideally even larger-scale databases than NIS could potentially provide more robust results. Third, the NIS provides exclusively in-hospital complication and mortality data. However, VTE and PE may occur after discharge. Consequently, in-hospital data may significantly underestimate true VTE and PE rates, as well as associated mortality, when the end point consists of outcomes with longer follow-up evaluation than the duration of the hospital stay.^9,21,25,26^ Fourth, multivariable adjustment relied on important patient and hospital characteristics available in the NIS. It is possible that other characteristics, such as personal or family history of VTE, use of thromboprophylaxis, and tumor status may have provided a better assessment of true VTE and PE rates. However, such additional characteristics were not available in the NIS and could not be considered.

## Conclusions

In the current study, RC, RN and PN predispose to higher VTE and PE rates than RP. Moreover, among RC and RN patients with either VTE or PE, mortality is substantially higher than among their VTE- or PE-free counterparts.

## References

[1] Geerts WH, Pineo GF, Heit JA (2004). Prevention of venous thromboembolism: the seventh ACCP conference on antithrombotic and thrombolytic therapy. Chest.

[2] Tikkinen KAO, Guyatt Gordon H (2020). Baseline risks of venous thromboembolism and major bleeding are crucial in decision-making on thromboprophylaxis.. Eur Urol.

[3] Tikkinen KAO, Cartwright R, Gould MK, et al. EAU Guidelines on Thromboprophylaxis in Urological Surgery. 2022. http://www.uroweb.org/guidelines/. Accessed 22 Feb 2023.

[4] Violette PD, Lavallée LT, Kassouf W, Gross PL, Shayegan B (2019). Canadian Urological Association guideline: perioperative thromboprophylaxis and management of anticoagulation. Can Urol Assoc J.

[5] Violette PD, Cartwright R, Briel M, Tikkinen KAO, Guyatt GH (2016). Guideline of guidelines: thromboprophylaxis for urological surgery. BJU Int.

[6] Agency for Healthcare Research and Quality. National Inpatient Sample (NIS) Healthcare Cost and Utilization Project (HCUP). Agency for Healthcare Research and Quality, Rockville, MD, USA, 2012. Retrieved 22 Feb 2023 at www.hcup-us.ahrq.gov/nisoverview.jsp.

[7] R: The R Project for Statistical Computing. Retrieved 12 June 2022 at https://www.r-project.org/.

[8] Björklund J, Stattin P, Rönmark E, Aly M, Akre O (2022). The 90-day cause-specific mortality after radical prostatectomy: a nationwide population-based study. BJU Int.

[9] McAlpine K, Breau RH, Mallick R (2017). Current guidelines do not sufficiently discriminate venous thromboembolism risk in urology. Urol Oncol Semin Orig Investig.

[10] Alberts BD, Woldu SL, Weinberg AC, Danzig MR, Korets R, Badani KK (2014). Venous thromboembolism after major urologic oncology surgery: a focus on the incidence and timing of thromboembolic events after 27,455 operations. Urology.

[11] Lyon TD, Tollefson MK, Shah PH (2018). Temporal trends in venous thromboembolism after radical cystectomy. Urol Oncol Semin Orig Investig.

[12] Tyson MD, Castle EP, Humphreys MR, Andrews PE (2014). Venous thromboembolism after urological surgery. J Urol.

[13] Ashrani AA, Gullerud RE, Petterson TM, Marks RS, Bailey KR, Heit JA (2016). Risk factors for incident venous thromboembolism in active cancer patients: a population-based case-control study. Thromb Res.

[14] Gregson J, Kaptoge S, Bolton T (2019). Cardiovascular risk factors associated with venous thromboembolism. JAMA Cardiol.

[15] Zhang Z, Lei J, Shao X (2019). Trends in hospitalization and in-hospital mortality from VTE, 2007 to 2016, in China. Chest.

[16] Zainfeld D, Djaladat H (2017). Enhanced recovery after urologic surgery: current applications and future directions. J Surg Oncol.

[17] Mabey E, Ismail S, Tailor T (2017). Improving venous thromboembolism risk assessment rates in a tertiary urology department. BMJ Open Qual.

[18] Schleyer AM, Robinson E, Dumitru R (2016). Preventing hospital-acquired venous thromboembolism: improving patient safety with interdisciplinary teamwork, quality improvement analytics, and data transparency. J Hosp Med.

[19] Goldsmith M, Whitelaw G, Cannaday DA (2008). VTE as a quality indicator. J Natl Compr Canc Net.

[20] Krimphove MJ, Reese S, Chen X (2020). Minimally invasive cancer surgery is associated with a lower risk of venous thromboembolic events. J Surg Oncol.

[21] Jordan BJ, Matulewicz RS, Trihn B, Kundu S (2017). Venous thromboembolism after nephrectomy: incidence, timing, and associated risk factors from a national multi-institutional database. World J Urol.

[22] Trinh VQ, Karakiewicz PI, Sammon J (2014). Venous thromboembolism after major cancer surgery: temporal trends and patterns of care. JAMA Surg.

[23] Kukreja JEB (2018). Perioperative venous thromboembolism in urologic oncology procedures, risk factors, and prevention. Curr Opin Urol.

[24] Tikkinen KAO, Craigie S, Agarwal A (2018). Procedure-specific risks of thrombosis and bleeding in urological cancer surgery: systematic review and meta-analysis. Eur Urol.

[25] Vandlac AA, Cowan NG, Chen Y (2014). Timing, incidence, and risk factors for venous thromboembolism in patients undergoing radical cystectomy for malignancy: a case for extended duration pharmacological prophylaxis. J Urol.

[26] Klaassen Z, Arora K, Goldberg H (2018). Extended venous thromboembolism prophylaxis after radical cystectomy: a call for adherence to current guidelines. J Urol.

